# Linking Challenge–Hindrance Stressors to Safety Outcomes and Performance: A Dual Mediation Model for Construction Workers

**DOI:** 10.3390/ijerph17217867

**Published:** 2020-10-27

**Authors:** Junwei Zheng, Xueqin Gou, Hongyang Li, Hong Xue, Hongtao Xie

**Affiliations:** 1 Faculty of Civil Engineering and Mechanics, Kunming University of Science and Technology, Kunming 650500, China; zjw1989@kust.edu.cn (J.Z.); gouxueqin@stu.kust.edu.cn (X.G.); 2 Business School, Hohai University, Nanjing 211100, China; lihy@hhu.edu.cn; 3 School of Civil Engineering and Transportation, South China University of Technology, Guangzhou 510641, China; 4 State Key Laboratory of Subtropical Building Science, South China University of Technology, Guangzhou 510641, China; 5 School of Management, Shandong University, Jinan 250100, China; xuehong@sdu.edu.cn; 6 Faculty of Management and Economics, Kunming University of Science and Technology, Kunming 650500, China

**Keywords:** challenge stressors, hindrance stressors, occupational injuries, attentiveness, task performance, construction workers

## Abstract

Occupational stressors have long been recognized as an important risk factor for injury accidents. The mechanisms underlying the relationships among challenge stressors, hindrance stressors, safety outcomes (occupational injuries), emotional experiences (attentiveness), and job performance (task performance) were investigated from the perspectives of the challenge–hindrance stress model and the conservation of resources theory. This study collected multi-source data over two timepoints for 105 safety supervisors and 379 construction workers in China. Results revealed that both challenge and hindrance stressors were positively related to occupational injuries, but only challenge stressors were positively associated with attentiveness. In addition, occupational injuries mediated the relationship between both challenge and hindrance stressors and task performance, while attentiveness mediated only the relationship between challenge stressors and task performance. These findings contribute to our knowledge of stress management in the construction project context and provide recommendations for stress management for front-line workers at construction sites.

## 1. Introduction

Occupational health and safety are a major focus in the practice of construction [1]. The International Labor Organization (ILO) reported that there are 374 million non-fatal job-related injuries every year [2]. The United Kingdom Health and Safety Executive (HSE) reported that although there was a downward trend in the self-reported and employer-reported rates of workplace non-fatal injuries from 2000 to 2019, non-fatal injuries nonetheless resulted in 4.7 million lost workdays in 2018–2019 [3]. According to the Statistical Communiqué of the People’s Republic of China on the 2019 National Economic and Social Development, the death toll from work-related accidents amounted to 29,519 [4]. Construction is one of the most dangerous industries, with a large number of accidents and higher than average incidence rates of work-related injuries [5]. Thus, because work-related accidents and injuries often occur on construction sites, the research and construction practice have paid more attention to the issues concerning occupational health and safety [6,7].

The evidence has shown that occupational stressors significantly increase one’s vulnerability to workplace accidents [1,8]. In the challenge–hindrance stress model (CHM) proposed by Cavanaugh et al. [9], occupational stressors are divided into two different categories, i.e., challenge stressors (CS) and hindrance stressors (HS). Challenge stressors refer to “manageable and challenging job-related demands or conditions like workload, time pressure, responsibilities, etc.” that relate to potential opportunities for personal growth, whereas hindrance stressors are “threatening and unmanageable job demands that include organization politics, role ambiguity, role conflict, job insecurity, etc.” that are associated with potential constraints on personal growth or achievement [9,10]. Many studies have investigated the differing effects of challenge–hindrance stressors on job outcomes. LePine et al. [11] stated that job performance is positively related to challenge stressors and negatively related to hindrance stressors. Podsakoff et al. [12] also showed that challenge stressors are positively associated with job outcome variables (e.g., job satisfaction and organizational commitment), whereas hindrance stressors have the opposite relationship to these job outcomes. Mazzola & Disselhorst [13] found a negative but nonsignificant relationship between challenge stressors and job performance, and nonsignificant findings were also mentioned in the research of Webster et al. [14]. Thus, the previous research offered mixed evidence for the impacts of challenge–hindrance stressors on job outcomes (e.g., job performance). The relationships between these two types of stressors and job outcomes such as performance remain an important but unanswered research question.

In the complex and uncertain environment of construction projects, construction workers need to perform specific tasks with multiple goals within a limited time period using finite resources [15,16]. The context of construction projects is characterized by long working hours and heavy workloads [17,18]. Construction professionals are likely to experience serious stressors, including challenge stressors, such as work overload, time pressure, and complexity, and hindrance stressors, such as role conflict and job insecurity. Although the prior research focused on the relationship between occupational stressors and workplace accidents (that would cause harm or damage) [19], there have been few studies on the impacts of occupational stressors on safety outcomes (e.g., occupational injuries or physical harm), and the studies that do exist have shown mixed results [1]. Specifically, Clarke [1] found a significant meta-analytic association between hindrance stressors and injuries (ρ = 0.14, 95% CI [0.05; 0.23]), and a nonsignificant correlation between challenge stressors and injuries (ρ = 0.02, 95% CI [–0.07; 0.10]). However, Nahrgang et al. [19] suggested that there is a significant meta-analytic correlation between challenge stressors (e.g., complexity) and injuries (ρ = 0.11, 95% CI [0.02; 0.21]). Moreover, the dangerous work environment of construction sites means that the workforce on site often experience work-related hazards that include exposure to extreme temperature, loud noise, and unsuitable lighting [20,21]. These dangerous conditions cause great stress as well as occupational accidents or injuries for construction workers (CWs) [22]. CWs are the front-line force employed to engage physical efforts and perform tasks in construction projects [23]. The task performance of CWs has direct impacts on project success with regard to quality, schedule, safety, cost [20,24], and profitability of construction firms [25]. Thus, these theoretical findings and construction practice both indicate that there is a value in further exploring the effects of occupational stressors on occupational injuries for CWs.

According to the transactional theory of stress [26], individuals respond to occupational stressors through cognitive appraisal and coping strategies. The cognitive appraisal depends on the types of stressors, i.e., whether the stressor is seen as potentially challenging or potentially threatening [26,27]. The outcome of the cognitive appraisal process influences both the emotional response and the coping strategy [11]. Moreover, according to the conservation of resources (COR) theory [28], individuals respond to a resource signal when gaining or depleting their personal resources; this involves the perceived availability of resources and the value and effort of investing resources. Thus, challenge–hindrance stressors induce the resource signal, because they deplete or force the investment of resources, and in turn influence the individual toward positive or negative coping strategies [12]. Combining these two theoretical viewpoints suggests that the occupational stressors for CWs might cause the cognitive appraisal and emotional responses and then influence their behavioral outcomes or performance.

**Figure 1:**
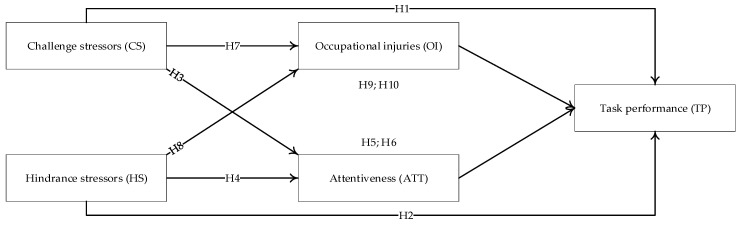
Conceptual model.

Furthermore, our study introduces attentiveness as the emotional reaction to occupational stressors. Previous studies provided evidence of the distinct relationships between challenge–hindrance stressors and behavioral outcomes or performance via emotions [29,30]. Thus, one of the purposes is to investigate whether challenge stressors and hindrance stressors would exhibit differential relationships with individual job performance through emotional responses for CWs. Further, by incorporating the COR theory and the transactional theory of stress, our study can also explore the cognitive processes linking challenge–hindrance stressors with individual performance. Another purpose is to address the underlying relationships between challenge–hindrance stressors and CWs’ task performance through safety outcomes (i.e., occupational injuries) in the context of construction projects. The proposed conceptual framework is summarized in Figure 1. Our study contributes to the body of knowledge by investigating the mediation effect of occupational injuries and attentiveness between challenge–hindrance stressors and task performance for construction workers in the context of construction projects. This study does so in three ways. First, it extends the application of CHM to safety management and stress management for CWs in construction projects. Using a multi-timepoint, multi-source research design, this study demonstrates whether CS and HS positively influence occupational injuries. Second, this study uses the theory of COR to explain the effects of job-related stressors on emotional experience, safety outcomes, and job performance. Third, this study not only differentiates between challenge stressors and hindrance stressors, but also examines the double-edged sword effects of challenge stressors on safety outcomes and positive emotions.

## 2. Theory and Hypotheses

### 2.1. Challenge–Hindrance Stressors and Task Performance

The CHM framework posits that occupational stressors are divided into two types, i.e., challenge and hindrance stressors, and that different stressors are associated with different job outcomes [9]. Challenge stressors, such as workload, time pressure, and high levels of responsibilities, are beneficial for personal growth and goal attainment, while hindrance stressors, such as role ambiguity, role conflict, and red tape, are barriers to the same. The growing body of research on the relationship between challenge–hindrance stressors and job outcomes suggests that CS are associated with desirable job-related behaviors or attitudes, including job satisfaction [12,14] and organizational commitment [12,31], whereas HS are associated with undesirable job-related behaviors or attitudes, such as withdrawal behavior [12] and turnover intention [14,31].

Not surprisingly, CS and HS have different effects on individual task performance. Task performance refers to the extent to which individuals successfully perform tasks related to their job description [32,33]. The positive relationship between CS and task performance and the negative relationship between HS and task performance have been identified by both meta-analysis [11] and empirical research [34,35]. This is because stressors such as a lack of performance guidance or the presence of too much red tape can lead individuals to feel exhausted or unsupported by the organization or their immediate supervisors [36]. By contrast, challenge stressors, such as high workload or high levels of responsibilities, may evoke positive individual attitudes such as motivation to achieve better performance [34].

Moreover, according to the transactional theory of stress [26], challenge stressors are viewed as opportunities for attainment, while hindrance stressors act as constraints on growth. Therefore, among CWs, we hypothesized that challenge stressors will generally facilitate performance while hindrance stressors will reduce performance. To wit:

**Hypothesis 1** **(H1).** *Challenge stressors have a positive relationship with task performance.*

**Hypothesis 2** **(H2).** *Hindrance stressors have a negative relationship with task performance.*

### 2.2. The Mediation Role of Attentiveness

Attentiveness refers to a positively valenced emotion [37] that includes feelings of alertness, determination, and attention [29]. It is a specific positive emotion focusing on engagement and task-related emotional responses [29,38]. Affective event theory (AET) is concerned with emotional reactions to events in the workplace and the subsequent behaviors [39]. Occupational stressors can be seen as affective events that can induce emotional reactions [29]. According to the appraisal and coping process developed by Lazarus and Folkman [26], stressors can be classified as “challenges” and “threats” that influence emotional experiences. In this paper, our analysis of affective states and the emotional reaction process created by challenge and hindrance stressors is informed by AET [39] and the transactional stress model [26]. Thus, the specific emotion of attentiveness reflecting high levels of pleasantness and engagement [40] is modeled to demonstrate the emotional reacting processes underlying stress appraisal.

Challenge stressors, when viewed as potential opportunities for personal growth or achievement, can facilitate success and trigger positive emotions [11]. The specific positive emotions are also responses to the opportunities for personal growth or goal achievement inherent in challenge stressors [29]. According to the COR theory, challenge stressors can enhance motivation and offset the potential negative impacts of stressors on the coping responses [41,42]. Thus, positive challenges can motivate individuals to dedicate to work and increase their engagement [43]. When coping with challenging job demands at construction sites, CWs may believe that they can deal with the job through personal effort. Challenge stressors can thus evoke feelings of confidence and trigger positive emotions. Goal attainment and personal growth are more likely when CWs pay their attention to the challenge stressor at hand, such as an impending deadline, or CWs are determined to complete the tasks at hand. Hence, we hypothesize:

**Hypothesis 3** **(H3).** *Challenge stressors have a positive relationship with attentiveness.*

Hindrance stressors viewed as potential threats to personal gain may trigger negative emotions or behaviors [9]. According to the transactional theory of stress [26], hindrance stressors represent threats or barriers to personal growth and the achievement of goals. When coping with hindrance stressors such as job insecurity, role conflict, and red tape, negative emotions such as anger or anxiety can arise in the workplace [29], and these hindering demands also decrease individual engagement [44,45]. This is because hindrance stressors are more likely to cause detrimental impacts on individual psychophysiological health to some extent [46] and lead to distress or dysfunctional effects on work-related outcomes [47]. For example, in the context of construction projects, uncertainty can be viewed as a potential threat to the completion of tasks. Dealing with hindrance stressors (e.g., task uncertainty) for CWs may increase their psychological strain [11,31] and reduce their work engagement [47]. Thus, we propose:

**Hypothesis 4** **(H4).** *Hindrance stressors have a negative relationship with attentiveness.*

Attentiveness is positively related to desirable job-related outcomes [29]. Attentiveness reflects high levels of engagement. [29,40] In other words, engaged CWs will be more attentive and absorbed in their tasks [48]. Thus, we expect to see a positive relationship between attentiveness and task performance. Furthermore, attentiveness can also be described as a condition of being fully engrossed in work, and this is a positive motivational and emotional state [27,48]. Based on the COR theory, challenge stressors may play a role in enhancing CWs’ motivation to be attentive, and in turn facilitate task performance, while hindrance stressors may deplete CWs’ resources (e.g., energy) to reduce their attentiveness and then decrease their task performance. We thus hypothesize:

**Hypothesis 5** **(H5).** *Attentiveness mediates the relationship between challenge stressors and task performance.*

**Hypothesis 6** **(H6).** *Attentiveness mediates the relationship between hindrance stressors and task performance.*

### 2.3. The Mediation Role of Occupational Injuries

Challenge stressors, such as work overload, time pressure, or complexity, can be viewed as opportunities to expand personal resources [26]. Prior studies showed that challenge stressors have positive impacts on safety behaviors, such as safety participation [1]. Thus, the possible impacts of challenge stressors on safety outcomes such as occupational injuries should also be taken into account. For example, performing complex tasks or meeting high job demands not only requires CWs to train and develop skills and knowledge for coping, but also extrinsically motivates CWs to work harder. Such pressure is a significant factor in occupational injuries [49]. High job task demands such as tight schedules contribute to the likelihood of incidents [50]. Moreover, poor working conditions such as working at height or exposure to inappropriate lighting can lead to a high likelihood of injury incidents [51]. Thus, we posit the hypothesis:

**Hypothesis 7** **(H7).** *Challenge stressors have a positive relationship with occupational injuries.*

Hindrance stressors, including role conflict and lack of job security, can seem threatening and difficult to overcome through the input of extra effort [26]. Previous studies suggested that there are negative relationships between hindrance stressors and job-related attitudes (e.g., job satisfaction) or behavioral outcomes (e.g., compliance), which in turn predict safety outcomes (e.g., occupational injuries) [1,52]. Clarke [1] has also stated that hindrance stressors may decrease individual motivation to comply with safety rules and to join in safety-specific activities. Such a tendency may then increase the likelihood of injuries. For example, role conflict may influence the willingness of CWs to carry out their responsibilities, or job insecurity may be detrimental to CW performance, increasing the likelihood of injuries at the construction site. Thus, we hypothesize:

**Hypothesis 8** **(H8).** *Hindrance stressors have a positive relationship with occupational injuries.*

Generally, the occurrence of occupational injuries at the construction site (i.e., physical injuries such as being struck by a moving object or slipping when lifting objects) may result in a reduction in the quality or schedule of performed tasks. According to the COR theory, occupational stressors deplete individual resources (e.g., physical resources); this depletion of resources increases the likelihood of injury incidents and, in turn, influences task performance. Therefore, we posit that occupational injuries play a mediation role in the relationships between challenge stressors, hindrance stressors, and task performance for CWs. To wit:

**Hypothesis 9** **(H9).** *Occupational injuries mediate the relationship between challenge stressors and task performance.*

**Hypothesis 10** **(H10).** *Occupational injuries mediate the relationship between hindrance stressors and task performance.*

## 3. Methods

### 3.1. Participants and Procedure

The data were collected through an on-site questionnaire survey of front-line construction workers and front-line safety supervisors working on the projects located in the Yunnan, Guizhou, Sichuan, and Jiangxi provinces of China. With the help of the associated construction team leader, paper questionnaires were distributed to the team’s workers and immediate supervisors. Before the survey, participants were informed that all the answers were guaranteed complete confidentiality and would be used only for this study to ensure anonymity and authenticity. To minimize the issue of common method variance, data collection was conducted in two stages and from two different sources. Specifically, during the first stage (timepoint 1), front-line construction workers were asked to report the challenge–hindrance stressors they experienced at work and their demographic information, such as age, gender, educational level, and type of job. One month later (timepoint 2), the same CWs were asked to report their level of attentiveness and occupational injuries over the past month; their immediate safety supervisors were invited to assess the CWs’ task performance.

At timepoint 1, the questionnaires were distributed among 704 construction workers working in 152 teams; 535 workers from 120 teams returned the survey, yielding a response rate of 82.3%. At timepoint 2, responses were received from 479 workers from 109 teams, yielding a response rate of 68.0%. After matching the data across the two timepoints and deleting invalid samples (mostly those with unvarying answers or missing data), the responses were finally obtained from 379 workers from 105 teams. Team sizes ranged from 3 to 6 (mean = 3.670, SD = 1.209). Regarding gender and age, the majority of the workers were male (94.4%) and the average age was 33.19 (SD = 8.313). Most of the CWs had a junior high school degree (64.9%); 48.8% were single and 51.2% were married; 32.1% were civil construction workers such as steel fixers and scaffolders, 32.9% were assembly workers such as welders and plumbers, 28.1% were decorators such as painters and plasterers, and 6.9% categorized their job as “other.”

### 3.2. Measures

The measurement items for variables involved in this study were originally developed in English. For this study, they were translated into Chinese and checked using the back-translation procedures recommended by Brislin [53]. After back-translation, construction professionals were invited to identify the appropriate items and propose reasonable advice for revision. The self-reported survey and supervisor-assessed survey were scored using 5-point Likert scales ranging from “1 = strongly disagree/never” to “5 = strongly agree/often”. The detailed items are listed in the Appendix A.

Challenge–hindrance stressors. CS and HS were measured with the scales developed by Cavanaugh et al. [9] to report CWs’ experiences on site ranging from 1 (produces no stress) to 5 (produces a great deal of stress). The CS scale included six items; an example item is “The volume of work that must be accomplished in the allotted time.” The Cronbach’s α for the CS scale was 0.863. The HS scale included five items; an example item is “The degree to which politics rather than performance affects organizational decisions.” The Cronbach’s α for the HS scale was 0.934.

Attentiveness. Attentiveness was measured using four items from the Positive and Negative Affect Schedule (PANAS) scale developed by Watson et al. [54] and adapted by Rodell and Judge [29]. Specifically, CWs were required to report the extent to which they experienced, in the past 30 days, four aspects of attentiveness: alert, attentive, strong, and determined. The Cronbach’s α for this scale was 0.831.

Occupational injuries. Occupational injuries were measured using the five items developed by Barling et al. [55]. CWs rated the frequency of the following injury incidents on site in the past 30 days: strains or sprains, cuts or lacerations, burns, bruises or contusions, and fractured bone. The Cronbach’s α for this scale was 0.863.

Task performance. Task performance was reported by direct supervisors using the five items developed by Williams and Anderson [33]. A sample item is “This construction worker has adequately completed assigned duties.” The Cronbach’s α for this scale was 0.803.

Control variables. This study controlled for the effects of the following demographic variables: age (year), gender (1 = male, 2 = female), educational level (1 = elementary school and below, 2 = junior high school, 3 = senior high school, 4 = junior college), marriage (1 = single, 2 = married), and job type (1 = civil construction worker (e.g., concrete worker, steel fixer, scaffolder, woodworker, etc.); 2 = assembly worker (e.g., welder, plumber, electrician, etc.); 3 = decorator (e.g., painter, plasterer, etc.); 4 = others).

## 4. Results

### 4.1. Preliminary Analysis

Confirmatory factor analysis (CFA). To examine the construct validity and discriminant validity of the focal variables, a series of confirmatory factor analyses (CFAs) were performed using MPLUS @ 7.0 [56]. As shown in Table 1, compared to the alternative models, the hypothesized five-factor model (challenge stressors, hindrance stressors, attentiveness, occupational injuries, and task performance) displayed an acceptable fit to the data (χ^2^_(198)_ = 596.649, χ^2^/df = 3.013, RMSEA (root-mean-square error of approximation) = 0.073, CFI (comparative fit index) = 0.920, TLI (Tacker–Lewis index) = 0.906, SRMR (standardized root mean squared residual) = 0.056). Other alternative models showed a poorer fit than the hypothesized five-factor model, which is evidenced by the increase in the enhanced values of RMSEA and SRMR and the decrease in the values of CFI and TLI. These CFA results demonstrate the construct validity and discriminant validity of the measures for the studied variables.

Descriptive statistics and correlation analysis. Table 2 presents the descriptive statistics (i.e., means and standard deviations (SD)), the average variance extracted (AVE), and the intercorrelations between the focal variables. The pattern of the correlations is in accordance with the hypothesized model. Specifically, the results indicate that challenge stressors are positively related to both attentiveness (r = 0.111, *p* < 0.05) and occupational injuries (r = 0.320, *p* < 0.001); hindrance stressors are positively associated with occupational injuries (r = 0.146, *p* < 0.01); attentiveness is positively correlated with task performance (r = 0.500, *p* < 0.001); occupational injuries are negatively correlated with task performance (r = −0.107, *p* < 0.05).

### 4.2. Testing of Hypotheses

Direct effects test. This study used SPSS @ 22.0 to test the research hypotheses. Hypothesis 1 and 2 suggest direct effects of CS and HS on task performance. The results of model 3, as shown in Table 3, indicate that CS and HS were insignificantly related to task performance (β = –0.002, *p* > 0.05; β = 0.037, *p* > 0.05). Thus, Hypotheses 1 and 2 were not supported.

Hypotheses 3 and 4 concern the direct effects of CS and HS on attentiveness. As shown in Table 3, after controlling for the demographic variables, CS were positively related to the CWs’ attentiveness (β = 0.103, *p* < 0.05, model 1), supporting Hypothesis 3. HS were positively but insignificantly related to the CWs’ attentiveness (β = 0.103, n.s., model 1), and so Hypothesis 4 was not supported.

Hypotheses 7 and 8 suggest the direct effects of CS and HS on occupational injuries. As shown in model 2 of Table 3, CS were positively related to occupational injuries (β = 0.513, *p* < 0.001), and HS were also positively related to occupational injuries (β = 0.430, *p* < 0.001, model 2). Thus, Hypotheses 7 and 8 were supported.

Furthermore, after controlling for challenge–hindrance stressors, attentiveness was positively related to task performance (β = 0.538, *p* < 0.001, model 4), while occupational injuries were negatively related to task performance (β = –0.068, *p* < 0.05, model 5).

Mediating effects test. Hypotheses 5, 6, 9 and 10 suggest mediation roles for attentiveness and injuries between CS, HS, and task performance. This study adopted the bootstrapping method and 95% confidence intervals (CIs) to examine the mediation effects of attentiveness and occupational injuries through the PROCESS macro in SPSS.

As shown in Table 4, the results indicate that attentiveness mediated the effect of CS on task performance (effect = 0.053, 95% CI [0.007, 0.102], model 6), but attentiveness played an insignificant mediation role in the relationship between HS and task performance (effect = −0.010, 95% CI [−0.059, 0.045], model 7). Thus, Hypothesis 5 was supported and Hypothesis 6 was not. Furthermore, occupational injuries mediated the effect of CS on task performance (effect = −0.030, 95% CI [−0.0553, −0.003], model 8), and the effect of HS on task performance (effect = −0.018, 95% CI [−0.047, −0.002], model 9). Therefore, Hypotheses 9 and 10 were supported.

## 5. Discussion

### 5.1. Major Findings

Challenge stressors and outcomes. Challenge stressors exhibited positive relationships with attentiveness and occupational injuries. These results demonstrate the multiple effects of challenge stressors on emotional responses and behavioral outcomes and support the viewpoints of Rosen et al. [57]. For example, a challenge stressor such as high workload may motivate individuals to work harder or to be more attentive to meet job demands, but may also cause physical fatigue or even more injuries. Furthermore, challenge stressors were found to have a positive indirect effect on task performance through the mediation of attentiveness but a negative indirect effect on task performance via occupational injuries. Both of these effects are consistent with the prior findings: individuals are likely to react to positive emotions by performing better, but to avoid situations that may lead them to experience negative events [29,58]. These findings suggest that some challenge stressors, such as job responsibilities, may improve attentiveness, but other challenge stressors, such as time pressure, may lead to injury accidents.

Hindrance stressors and outcomes. Hindrance stressors exhibited a positive relationship with occupational injuries, but failed to exhibit a significant relationship with attentiveness. The relationship between hindrance stressors and task performance can be explained by an indirect effect via occupational injuries. Furthermore, we did not find a statistically significant relationship between hindrance stressors and attentiveness. That is to say, CWs’ positive emotions or attention do not appear to vary as a function of the level of hindrance stressors, such as role conflict or job insecurity. No mediation role was found for attentiveness in the association between hindrance stressors and task performance. A possible reason for this is that hindrance stressors are perceived as a threat and individuals are likely to respond to hindrance stressors with negative emotions such as anxiety [9,29]. The negative emotional reactions would then cause problem behaviors; hindrance stressors are therefore likely to be associated with negative emotional experiences and passive behaviors in the workplace and then lead to injury accidents. Additionally, although these findings for hindrance stressors were inconsistent with expectations, they still highlight the difference between hindrance stressors and challenge stressors and distinguish the relationships between distinct stressors and individual task performance.

### 5.2. Theoretical Implications

This study has three key theoretical implications. Firstly, it supports the application of the challenge–hindrance framework for safety management in the context of construction projects for CWs. The previous literature focused on the stressors of construction project managers (C-PMs) [16], who play a critical role in achieving project success. Moreover, Leung and colleagues [20,25] have also investigated different types of stressors, including organizational, physical, personal stressors for CWs, and the impacts of stressors on safety outcomes (e.g., safety behaviors, injury incidents). Unlike the previous work on job stressors for C-PMs or CWs [16,20,25], this study extended and explored distinct categories of stressors and their effects on safety outcomes for CWs, such as hindrance stressors (e.g., role conflict, job insecurity). Besides, although previous studies found that both CS and HS may relate to the same outcome in opposite directions [11,59], our findings indicate that this is not always the case. By examining the effects of challenge–hindrance stressors on occupational injuries, this study found that both CS and HS promote occupational injuries among CWs. These findings highlight the importance of considering challenge–hindrance stressors for the safety management and stress management of front-line workers in construction projects.

Secondly, this study enhances the explanation given by the COR theory for the effects of stressors in the context of safety management on construction projects. The existing literature has provided evidence that the variations in job performance and satisfaction induced by job stressors can be attributed to a high level of strain [12] or to a perceived low level of justice in the response to stressors [42]. However, from the perspective of the COR theory, in addition to the action signal (e.g., strain) and the value signal (e.g., perception of justice), there is another signal, that of resource availability, which should also be examined: specifically, whether there are more resources available for responding to stressors. In this study, we found that CS are beneficial for improving the emotional resource—attentiveness—that in turn facilitates task performance. These results not only enhance the explanation given by the COR theory for the role of availability of resources in influencing job stressors, but also indicate the positive aspect (e.g., challenge stressors) of the double-edged sword of job stressors. Specifically, these findings supplement the results of Leung et al. [25,52] who found that organizational stressors are significantly and positively related to emotional stress for CWs.

Thirdly, this study contributes to the knowledge of double-edged effects of challenge stressors themselves. Although the empirical findings show that challenge stressors are likely to increase attentiveness and improve job performance in agreement with the prior studies [29], this kind of stressor is nonetheless unable to affect safety outcomes [1] and is likely to lead to an increase in injury accidents in the workplace. Thus, with regard to high job demands in the project context, including time pressure, complexity, etc., extra efforts should be invested to maintain job-related outcomes and safety. This “trade-off” strategy needs to be considered to simultaneously ensure project completion and safety in project management.

### 5.3. Practical Implications

This study has several implications for project practice. First, the findings reveal that stressful work can potentially trigger injury accidents. Construction organizations that are concerned with decreasing the occurrence of injury accidents would benefit from focusing on the level of challenge–hindrance stressors, as these can induce injury accidents among front-line workers. Organizations could manage or control hindrance stressors by reducing unnecessary processes and meetings (i.e., by decreasing the red tape) and by providing clear instructions (i.e., by reducing role ambiguity). However, as shown in the study, too many challenge stressors (e.g., overload, job demands) also increase the risk of injury for construction workers. Hometown groups for CWs may be a good approach to establish and develop informal relationships to increase coworker support, exchange work experiences, and offer necessary assistance [60]. The supervisor on site could assign the CWs to relevant working teams to assist each other and encourage the mutual observance or supervision for safety during the task [20].

Additionally, given the different effects of challenge and hindrance stressors on attentiveness, project managers should not only reduce the presence of hindrance stressors (e.g., red tape, organizational politics,) but also establish norms to set an appropriate level of challenge stressors on site. Although it is impossible to completely eliminate job stress, moderate challenge stressors can help CWs to focus on performing their tasks. Construction organizations can establish programs to train CWs to learn how to balance challenge stressors and hindrance stressors, and CWs can be trained to regulate their emotions in response to challenge stressors, so that they can increase their attentiveness and become more productive. Moreover, unsafe or stressful working environment can make it difficult for CWs to focus on their work. Construction organizations could consider conducting regular tests to train and guide CWs to understand job demands, observe safety regulations, and update knowledge and skills [20]. Stress reduction interventions like relaxation exercises can also be considered to help CWs to overcome and reduce the stress stemming from the poor working environment [61].

### 5.4. Limitations and Future Directions

There are several potential limitations to this study. Firstly, a sample of construction workers (CWs) was used in this study to investigate safety management. A sample of construction project managers (C-PMs) has been focused on previously [16], and there may be differences in the stressors of CWs and C-PMs. Future research could compare the stressors experienced by CWs and C-PMs and explore their different effects on work outcomes and the mechanisms by which they exert those effects.

Secondly, this study only posited a linear relationship between challenge–hindrance stressors and project performance. Future research may seek to establish whether there may be a curvilinear relationship instead. For example, the peak level of CS may result in desirable work-related outcomes, but CS beyond that may lead to an increase in negative outcomes [13].

Thirdly, this study only explored the influencing path from challenge–hindrance stressors to project performance, with occupational injuries and attentiveness playing mediation roles. Future research could attempt to explore boundary conditions, such as conscientiousness [10] and leadership [42]. It is possible to investigate under different conditions the variations in strength and direction of the relationships between CS, HS, and safety outcomes or individual performance.

## 6. Conclusions

CWs work in a complex physical environment amid a range of potential hazards, including exposure to extreme temperatures, loud noises, unsuitable lighting, etc. This directly impacts their stress levels, emotional experiences, risk of injury, and job performance. Based on the CHM and COR theories, this study investigated the differences in CS and HS and their effects on CWs in the construction project context and explored the direct relationships between CS, HS, and task performance and the mediating role of occupational injuries and attentiveness. This study did so through scientific research methodologies that included multi-source and multi-timepoint data collection and statistical analyses, such as regression analysis and bootstrapping. The results indicated that occupational injuries are increased by both CS and HS and that the attentiveness of CWs is improved by CS. The task performance of CWs is hampered by both CS and HS through increased injuries, but is facilitated by CS through improved attentiveness. These findings highlight the different effects of CS and HS in the construction project context and explain the effects of CS and HS on job performance from the CHM and COR perspectives. With regard to the distinctive working environment at sites, project managers should be aware of the stressors affecting CWs and, in particular, should identify and control hindrance stressors and train CWs in skills of emotional regulation to improve occupational health and safety and facilitate task performance.

## Figures and Tables

**Table 1:** Results of the confirmatory factor analysis.

Models	χ^2^(df)	χ^2^/df	RMSEA	CFI	TLI	SRMR
Five-factor: CS, HS, AT, OI, TP	596.649(198)	3.013	0.073	0.920	0.906	0.056
Four-factor: CS + HS, AT, OI, TP	1935.521(203)	9.535	0.150	0.651	0.603	0.124
Three-factor: CS + HS, AT + OI, TP	2273.161(206)	11.035	0.163	0.584	0.533	0.132
Two-factor: CS + HS + AT, OI + TP	3078.951(208)	14.803	0.191	0.422	0.358	0.165
Single-factor: CS + HS + AT + OI + TP	4064.973(209)	19.364	0.221	0.223	0.142	0.215

Note: CS = challenge stressors; HS = hindrance stressors; AT = attentiveness; OI = occupational injuries; TP = task performance; RMSEA = root-mean-square error of approximation; CFI = comparative fit index; TLI = Tacker–Lewis index; SRMR = standardized root mean squared residual.

**Table 2:** Descriptive statistics and intercorrelations between measures.

Variables	Mean	SD	1	2	3	4	5
1. CS (T1)	4.341	0.912	(0.816)				
2. HS (T1)	1.753	0.697	−0.268 ***	(0.889)			
3. AT (T2)	2.036	0.710	0.111 *	0.018	(0.745)		
4. OI (T2)	2.777	1.200	0.320 ***	0.146 **	−0.008	(0.755)	
5. TP (T2)	2.264	0.748	−0.013	0.045	0.500 ***	−0.107 *	(0.898)

Note: The number of samples = 379. CS = challenge stressors; HS = hindrance stressors; AT = attentiveness; OI = occupational injuries; TP = task performance. Values in the parenthesis are the square roots of the AVE. *** *p* < 0.001, ** *p* < 0.01, * *p* < 0.05.

**Table 3:** Results of the regression analysis.

Variables	AT (T2)	OI (T2)	TP (T2)		
	Model 1	Model 2	Model 3	Model 4	Model 5
Control variables					
Age	−0.003 (0.005)	−0.007 (0.008)	0.000 (0.005)	0.002 (0.005)	0.000 (0.005)
Gender	−0.169 (0.163)	−0.046 (0.255)	−0.235 (0.173)	−0.144 (0.149)	−0.238 (0.172)
Education	−0.069 (0.051)	−0.016 (0.081)	0.006 (0.055)	0.044 (0.047)	0.005 (0.054)
Marriage	0.115 (0.085)	0.043 (0.133)	−0.046 (0.090)	−0.108 (0.078)	−0.043 (0.090)
Job types	0.015 (0.037)	−0.014 (0.058)	−0.044 (0.039)	−0.052 (0.034)	−0.045 (0.039)
Independent Variables					
CS (T1)	0.103 * (0.042)	0.513 *** (0.066)	−0.002 (0.045)	−0.057 (0.039)	0.033 (0.048)
HS (T1)	0.017 (0.054)	0.430 *** (0.085)	0.046 (0.058)	0.037 (0.050)	0.076 (0.059)
Mediators					
AT (T2)				0.538 *** (0.048)	
OI (T2)					−0.068 * (0.035)
R^2^	0.025	0.162	0.012	0.266	0.022
F	1.363	10.214 ***	0.620	16.659 ***	1.102

Note: CS = challenge stressors; HS = hindrance stressors; AT = attentiveness; OI = occupational injuries; TP = task performance. *** *p* < 0.001, * *p* < 0.05.

**Table 4:** Results of the mediation testing using PROCESS (Bootstrap = 5000).

Paths	Effect	SE	95% CI	
			LL	UL
Model 6: Challenge stressors → Attentiveness → Task performance	0.053	0.024	0.007	0.102
Model 7: Hindrance stressors → Attentiveness → Task performance	−0.010	0.027	−0.059	0.045
Model 8: Challenge stressors → Injuries → Task performance	−0.030	0.014	−0.053	–0.003
Model 9: Hindrance stressors → Injuries → Task performance	−0.018	0.011	−0.047	–0.002

## Appendix A

**Detailed Items**

Challenge–hindrance stressors [Please report the stress experience on site for the past 30 days ranging from 1 (produces no stress) to 5 (produces a great deal of stress).]

Challenge stressors

1. The number of projects and or assignments I have
2. The amount of time I spend at work
3. The volume of work that must be accomplished in the allotted time
4. Time pressures I experience
5. The amount of responsibility I have
6. The scope of responsibility my position entails

Hindrance stressors

1. The degree to which politics rather than performance affects organizational decisions
2. The inability to clearly understand what is expected of me at work
3. The amount of red tape I need to go through to get my job done
4. The lack of job security I have
5. The degree to which my career seems “stalled”

Attentiveness [In the past 30 days’ work on site, I felt… ranging from 1 (never) to 5 (often).]

1. Alert
2. Attentive
3. Strong
4. Determined

Occupational injuries [How many times have you suffered from the following injuries in the last 30 days’ work? 1 = never; 5 = often.]

1. Strains or sprains
2. Cuts or lacerations
3. Burns
4. Bruises or contusions
5. Fractured bone

Task performance [Please assess the work-related task performance of a construction worker in your team in the past 30 days ranging from 1 (strongly disagree) to 5 (strongly agree).]

1. This construction worker adequately completed the assigned duties.
2. This construction worker fulfilled the responsibilities specified in his/her job description.
3. This construction worker performed the tasks that are expected of him/her.
4. This construction worker met the formal performance requirements of the job.
5. This construction worker engaged in the activities that will directly affect his/her performance evaluations.

## References

[1] Clarke S. (2012). The effect of challenge and hindrance stressors on safety behavior and safety outcomes: A meta-analysis. J. Occup. Health Psychol..

[2] International Labour Organization Safety and Health at Work. https://www.ilo.org/global/topics/safety-and-health-at-work/lang--en/index.htm.

[3] Health and Safety Executive Non-fatal Injuries at Work in Great Britain. https://www.hse.gov.uk/statistics/causinj/index.htm.

[4] National Bureau of Statistics of China Statistical Communiqué of the People’s Republic of China on the 2019 National Economic and Social Development. http://www.stats.gov.cn/english/PressRelease/202002/t20200228_1728917.html.

[5] Fang D., Wu H. (2013). Development of a safety culture interaction (SCI) model for construction projects. Saf. Sci..

[6] Guo B.H.W., Yiu T.W., González V.A. (2016). Predicting safety behavior in the construction industry: Development and test of an integrative model. Saf. Sci..

[7] Lingard H.C., Cooke T., Blismas N. (2010). Safety climate in conditions of construction subcontracting: A multi-level analysis. Constr. Manag. Econ..

[8] Trimpop R., Kirkcaldy B., Athanasou J., Cooper C. (2000). Individual differences in working hours, work perceptions and accident rates in veterinary surgeries. Work Stress.

[9] Cavanaugh M.A., Boswell W.R., Roehling M.V., Boudreau J.W. (2000). An empirical examination of self-reported work stress among U.S. managers. J. Appl. Psychol..

[10] Abbas M., Raja U. (2019). Challenge-Hindrance Stressors and Job Outcomes: The Moderating Role of Conscientiousness. J. Bus. Psychol..

[11] Lepine J.A., Podsakoff N.P., Lepine M.A. (2005). A meta-analytic test of the challenge stressor–hindrance stressor framework: An explanation for inconsistent relationships among stressors and performance. Acad. Manag. J..

[12] Podsakoff N.P., LePine J.A., LePine M.A. (2007). Differential challenge stressor-hindrance stressor relationships with job attitudes, turnover intentions, turnover, and withdrawal behavior: A meta-analysis. J. Appl. Psychol..

[13] Mazzola J.J., Disselhorst R. (2019). Should we be “challenging” employees?: A critical review and meta-analysis of the challenge-hindrance model of stress. J. Organ. Behav..

[14] Webster J.R., Beehr T.A., Christiansen N.D. (2010). Toward a better understanding of the effects of hindrance and challenge stressors on work behavior. J. Vocat. Behav..

[15] Munns A., Bjeirmi B. (1996). The role of project management in achieving project success. Int. J. Proj. Manag..

[16] Leung M., Chan Y.-S., Yu J. (2009). Integrated model for the stressors and stresses of construction project managers in Hong Kong. J. Constr. Eng. Manag..

[17] Liu J.Y., Low S.P. (2011). Work-family conflicts experienced by project managers in the Chinese construction industry. Int. J. Proj. Manag..

[18] Turner R., Huemann M., Keegan A. (2008). Human resource management in the project-oriented organization: Employee well-being and ethical treatment. Int. J. Proj. Manag..

[19] Nahrgang J.D., Morgeson F.P., Hofmann D.A. (2011). Safety at work: A meta-analytic investigation of the link between job demands, job resources, burnout, engagement, and safety outcomes. J. Appl. Psychol..

[20] Leung M., Liang Q., Olomolaiye P. (2016). Impact of job stressors and stress on the safety behavior and accidents of construction workers. J. Manag. Eng..

[21] Hoonakker P., van Duivenbooden C. (2010). Monitoring working conditions and health of older workers in Dutch construction industry. Am. J. Ind. Med..

[22] Gouett M.C., Haas C.T., Goodrum P.M., Caldas C.H. (2011). Activity analysis for direct-work rate improvement in construction. J. Constr. Eng. Manag..

[23] Fung I.W.H., Tam C.M., Tung K.C.F., Man A.S.K. (2005). Safety cultural divergences among management, supervisory and worker groups in Hong Kong construction industry. Int. J. Proj. Manag..

[24] Djebarni R. (1996). The impact of stress in site management effectiveness. Constr. Manag. Econ..

[25] Leung M., Chan Y.-S., Yuen K.-W. (2010). Impacts of stressors and stress on the injury incidents of construction workers in Hong Kong. J. Constr. Eng. Manag..

[26] Lazarus R.S., Folkman S. (1984). Stress, Appraisal, and Coping.

[27] Kahn W.A. (1990). Psychological conditions of personal engagement and disengagement at work. Acad. Manag. J..

[28] Halbesleben J.R.B., Neveu J.P., Paustian-Underdahl S.C., Westman M. (2014). Getting to the “COR”: Understanding the role of resources in conservation of resources theory. J. Manag..

[29] Rodell J.B., Judge T.A. (2009). Can “good” stressors spark “bad” behaviors? The mediating role of emotions in links of challenge and hindrance stressors with citizenship and counterproductive behaviors. J. Appl. Psychol..

[30] Pearsall M.J., Ellis A.P.J., Stein J.H. (2009). Coping with challenge and hindrance stressors in teams: Behavioral, cognitive, and affective outcomes. Organ. Behav. Hum. Decis. Process..

[31] Boswell W.R., Olson-Buchanan J.B., LePine M.A. (2004). Relations between stress and work outcomes: The role of felt challenge, job control, and psychological strain. J. Vocat. Behav..

[32] Campbell J.P., Rumsey M.G., Walker C.B., Harris J.H. (1994). Alternative models of job performance and their implications for selection and calssification. Personnel Selection and Classification.

[33] Williams L.J., Anderson S.E. (1991). Job satisfaction and organizational commitment as predictors of organizational citizenship and in-role behaviors. J. Manag..

[34] Lin W., Ma J., Wang L., Wang M. (2015). A double-edged sword: The moderating role of conscientiousness in the relationships between work stressors, psychological strain, and job performance. J. Organ. Behav..

[35] Wallace J.C., Edwards B.D., Arnold T., Frazier M.L., Finch D.M. (2009). Work stressors, role-based performance, and the moderating influence of organizational support. J. Appl. Psychol..

[36] Haar J.M. (2006). Challenge and hindrance stressors in New Zealand: Exploring social exchange theory outcomes. Int. J. Hum. Resour. Manag..

[37] Watson D. (2000). Mood and Temprament.

[38] Weick K.E., Roberts K.H. (1993). Collective mind in organizations: Heedful interrelating on flight decks. Adm. Sci. Q..

[39] Weiss H.M., Cropanzano R., Staw B.M., Cummings L.L. (1996). Affective events theory: A theoretical discussion of the structure, causes and consequences of affective experiences at work. Research in Organizational Behavior.

[40] Watson D., Tellegen A. (1985). Toward a consensual structure of mood. Psychol. Bull..

[41] Crawford E.R., LePine J.A., Rich B.L. (2010). Linking job demands and resources to employee engagement and burnout: A theoretical extension and meta-analytic test. J. Appl. Psychol..

[42] Zhang Y., Lepine J.A., Buckman B.R., Wei F. (2014). It’s not fair...or is it? The role of justice and leadership in explaining work stressor-job performance relationships. Acad. Manag. J..

[43] Jiang Q., Lee H., Xu D. (2020). Challenge stressors, work engagement, and affective commitment among Chinese public servants. Public Pers. Manag..

[44] Li P., Taris T.W., Peeters M.C.W. (2020). Challenge and hindrance appraisals of job demands: One man’s meat, another man’s poison?. Anxiety Stress Coping.

[45] Wilson C.A., Britt T.W. (2020). Living to work: The role of occupational calling in response to challenge and hindrance stressors. Work Stress.

[46] Wu H., Qiu S., Dooley L.M., Ma C. (2020). The relationship between challenge and hindrance stressors and emotional exhaustion: The moderating role of perceived servant leadership. Int. J. Environ. Res. Public Health.

[47] Babakus E., Yavas U., Karatepe O.M. (2017). Work engagement and turnover intentions: Correlates and customer orientation as a moderator. Int. J. Contemp. Hosp. Manag..

[48] Shantz A., Alfes K., Truss C., Soane E. (2013). The role of employee engagement in the relationship between job design and task performance, citizenship and deviant behaviours. Int. J. Hum. Resour. Manag..

[49] Sakurai K., Nakata A., Ikeda T., Otsuka Y., Kawahito J. (2013). How do employment types and job stressors relate to occupational injury? A cross-sectional investigation of employees in Japan. Public Health.

[50] Goldenhar L.M., Williams L.J., Swanson N.G. (2003). Modelling relationships between job stressors and injury and near-miss outcomes for construction labourers. Work Stress.

[51] Leung M., Chan I.Y.S., Yu J. (2012). Preventing construction worker injury incidents through the management of personal stress and organizational stressors. Accid. Anal. Prev..

[52] Clarke S. (2010). An integrative model of safety climate: Linking psychological climate and work attitudes to individual safety outcomes using meta-analysis. J. Occup. Organ. Psychol..

[53] Brislin R.W. (1970). Back-translation for cross-cultural research. J. Cross. Cult. Psychol..

[54] Watson D., Clark L.A., Tellegen A. (1988). Development and validation of brief measures of positive and negative affect: The PANAS scales. J. Pers. Soc. Psychol..

[55] Barling J., Loughlin C., Kelloway E.K. (2002). Development and test of a model linking safety-specific transformational leadership and occupational safety. J. Appl. Psychol..

[56] Muthén L.K., Muthén B.O. (2012). Mplus User’s Guide.

[57] Rosen C.C., Chang C.-H., Djurdjevic E., Eatough E., Perrewé P.L., Ganster D.C. (2010). Occupational stressors and job performance: An updated review and recommendations. New Developments in Theoretical and Conceptual Approaches to Job Stress (Research in Occupational Stress and Well Being, Vol.8).

[58] Lazarus R.S. (1991). Progress on a cognitive-motivational-relational theory of emotion. Am. Psychol..

[59] Liu C., Liu Y., Mills M.J., Fan J. (2013). Job stressors, job performance, job dedication, and the moderating effect of conscientiousness: A mixed-method approach. Int. J. Stress Manag..

[60] Canales A.R., Arbelaez M., Vasquez E., Aveiga F., Strong K., Walters R., Jaselskis E.J., Jahren C.T. (2009). Exploring training needs and development of construction Language courses for American supervisors and Hispanic craft workers. J. Constr. Eng. Manag..

[61] Moraska A., Pollini R.A., Boulanger K., Brooks M.Z., Teitlebaum L. (2010). Physiological adjustments to stress measures following massage therapy: A review of the literature. Evid.-Based Complement. Altern. Med..

